# Microarray and bioinformatic analysis reveal the parental genes of m6A modified circRNAs as novel prognostic signatures in colorectal cancer

**DOI:** 10.3389/fonc.2022.939790

**Published:** 2022-07-29

**Authors:** Wenken Liang, Liyuan Deng, Chune Mo, Wei Chen, Yu Sha, Jianling Shi, Xianliang Hou, Yuping Zhang, Min Yang, Minglin Ou

**Affiliations:** ^1^ Central Laboratory, Guangxi Health Commission Key Laboratory of Glucose and Lipid Metabolism Disorders, The Second Affiliated Hospital of Guilin Medical University, Guilin, China; ^2^ College of Life Science, Guangxi Normal University, Guilin, China; ^3^ Department of Hematology, The Second Affiliated Hospital of Guilin Medical University, Guilin, China; ^4^ Gastrointestinal Surgery, The Second Affiliated Hospital of Guilin Medical University, Guilin, China; ^5^ Department of general medicine, Guilin Medical University, Guilin, China; ^6^ Department of Rheumatology and Immunology, The Affiliated Hospital of Guilin Medical University, Guilin, China

**Keywords:** TCGA, CRC, m6A, circRNAs, prognostic signature

## Abstract

**Background:**

Accumulating evidences have revealed that the abnormal N6-methyladenosine (m6A) modification is closely associated with the occurrence, development, progression and prognosis of cancer. It is noteworthy that m6A modification is widely existed in circRNAs and found its key biological functions in regulating circRNAs metabolism. However, the role of m6A modified circRNAs in colorectal cancer (CRC) remains unknown. To better understand the role of circRNAs in the pathogenesis of CRC, we focus on the relationship between m6A-modified circRNAs and their parental genes.

**Methods:**

Arraystar m6A-circRNA epitranscriptomic microarray was used to identify differentially m6A modified circRNAs between CRC and the control group. In addition, TCGA-COAD and GSE106582 cohort were used to identify differentially expressed mRNAs. In this study, we screened the parental genes for which both circRNAs and mRNAs were down-regulated further to analyze, including gene expression, survival prognosis, enrichment analysis. Additionally, Western Blotting was used to further validate the role of the parental gene in CRC.

**Results:**

We found that 1405 significantly downregulated circRNAs in CRC by our microarray data. Moreover, we obtained 113 parental genes for which both circRNAs and mRNAs were down-regulated to analyze the relationship with the prognosis of CRC based on TCGA-COAD cohort. And we identified nine potential prognostic genes, including *ABCD3*, *ABHD6*, *GAB1*, *MIER1*, *MYOCD*, *PDE8A*, *RPS6KA5*, *TPM1* and *WDR78*. And low expression of these genes was associated with poor survival prognosis of the patients with CRC. In addition, we found that TPM1 is downregulated in CRC by western blotting experiment. And the calcium-signaling pathway may involve the process of the CRC progression.

**Conclusions:**

We identified nine potential prognostic genes, after analyzed the relationship between the parental genes of m6A modified circRNAs and the progression of CRC. Above all, our study further validated TPM1 can serve as a potentail signature for CRC patients.

## Introduction

Colorectal cancer (CRC) is the third most commonly diagnosed cancer, and more than 1.9 million new CRC (including anus) cases and 935,000 deaths were estimated to occur in 2020, representing about one in 10 cancer cases and deaths (1). In the past few decades, the incidence and mortality rates of CRC have been increasing in China (2). In 2020, newly diagnosed CRC cases in China accounted for 28.8% of all new cases worldwide, and CRC-associated deaths accounted for 30.6% of all CRC-related deaths worldwide (3). It is noteworthy that most CRC patients are diagnosed in the terminal stage due to the lack of obvious symptoms. Thus, it is valuable to explore the molecular pathogenesis of CRC and identify credible prognostic signatures.

N6-methyladenosine (m6A) is the most prevalent modification of messenger RNA in mammals (4). And a study has reported that abnormal m6A modification is closely associated with the occurrence, development, progression and prognosis of cancer (5). Additionally, there is growing evidence that the dysregulation of circular RNAs (circRNAs) could play critical roles in the initiation and progression of cancer (6, 7). Recently, many researchers have found that m6A modified circRNAs could serve as biomarkers of cancer, such as colorectal cancer (8), gastric cancer (9) and hepatocellular carcinoma (10, 11). And it is necessary further to explore the role of m6A modified circRNAs in the pathogenesis of cancer.

Recently, some studies have revealed that circRNAs regulate the transcription of their parental genes by interaction with the transcriptional complexes (12–14). For example, a study has reported that *FLI1* exonic circular RNA utilizes a positive feedback mechanism to activate the transcription of *FLI1* by inducing DNA hypomethylation in CpG islands of the promoter, which drive the metastasis of breast cancer (15). And the study also implies that circRNAs resulting in the abnormal expression of their parental genes play a critical role in the progression of cancer. It is noteworthy that m6A modification is widely existed in circRNAs and found its key biological functions in regulating circRNA metabolism (16). Previous studies have revealed that different proportions of chemical modification of the same circRNAs may result in different cell outcomes (17, 18). To better understand the role of circRNAs in the pathogenesis of CRC, we focus on the relationship between the parental genes of m6A-modified circRNAs and the CRC progression.

In this study, we aimed to establish the expression profile of CRC through human m6A-circRNA Epitranscriptomic Microarray. And differentially m6A-modified circRNAs between CRC and the control group were identified by filtering with the fold change and p-value. And the clinical information of mRNAs generated from the parental genes of circRNAs were downloaded from TCGA database to further analyze, including gene expression analysis and survival prognosis. The flowchart (19, 20) of this study is shown in **Figure S1**.

## Materials and methods

### Clinical tissue samples

All clinical samples of CRC patients were obtained from the Second Affiliated Hospital of Guilin Medical College in China, including CRC samples (n = 14) and paracancer tissues (n = 12). In the study, paracancer tissues were used as a control group. The ethics committee of the Second Affiliated Hospital of Guilin Medical College has authorized this study on September 24,2021. And all CRC patients have signed informed consent forms. After surgery, all samples were quickly stored at -80°C.

### RNA isolation and m6A immunoprecipitation

TRIzol reagent (Sigma) was used to extract total RNA from each sample. And the NanoDrop ND-1000 was used to determine the concentrations of total RNA. Moreover, RNA integrity and gDNA contamination were assessed by Denaturing Agarose Gel Electrophoresis. We took equal amounts of total RNA from 10 CRC samples and prepared a 5 ug pooled sample as the tumor group. Similarly, total RNA from 8 paracancer samples was also prepared a 5 ug pooled sample as the control group. About 5 ug of total RNA and m6A spike-in control mixture were appended into 300 ul buffer (1×) including 2 ug anti-m6A rabbit polyclonal antibody (Synaptic Systems), and the reaction was incubated for 2 hours at 4°C. About 20 ul Dynabeads™ M-280 Sheep Anti-Rabbit IgG (Invitrogen) suspension was sealed at 4°C for 2 hours with 0.5% BSA. Washed three times with 300 ul IP buffer (1×), and resuspended in the total RNA-antibody mixture. Subsequently, the total RNA-antibody mixture was incubated with m6A-antibody beads at 4°C for 2 hours. After washing and elution, “IP” RNAs were obtained by acid phenol-chloroform and ethanol precipitated.

### RNase R treatment, labeling and hybridization

The “IP” RNAs were digested with RNase R (Epicentre) to eliminate linear RNAs and enrich circRNAs. We added equal amount of calibration spike-in control RNA into the enriched “IP” RNAs, amplified and labeled with Cy5 using Arraystar Super RNA Labeling Kit (Arraystar, AL-SE-005). Subsequently, the synthesized circRNAs were purified by RNeasy Mini Kit (QIAGEN, 74105). We also used NanoDrop ND-1000 to measure the concentration and specific activity of the purified circRNAs. 5 ul Blocking Agent (10×) and 1ul Fragmentation Buffer (25×) were used to fragment the circRNA, heating at 60℃ for 30min, and the fragmented circRNAs were combined with 25 ul Hybridization buffer (2×). 50 ul hybridization solution was added into the gasket slide and assembled to the m6A-circRNA Epitranscriptomic Microarray slide. The slides were incubated for 17 hours at 65°C in an Agilent Hybridization Oven. The hybridized arrays were washed, fixed and scanned using an Agilent Scanner G2505C.

### Data analysis

Agilent Feature Extraction software (version 11.0.1.1) was used to extract the data of the array images. Raw intensities of “IP” (Cy5-labelled) were normalized as the relative intensities through the RNA spike-in calibration controls. The normalized intensities of “IP” represent the “m6A quantity” of the transcript of the tumor and the control group. Differentially m6A-modified circRNAs between the tumor group and the control group were identified through filtering with fold change (FC) and statistical significance cutoffs. The R software was used to perform Hierarchical Clustering.

### Public databases and analysis

RNA sequencing data and clinical information of colon adenocarcinoma (COAD) patients were downloaded from The Cancer Genome Atlas (TCGA) database (https://portal.gdc.cancer.gov/) (21). A total of 506 COAD patients were explored differentially expressed mRNAs (DEmRNAs). In addition, the GSE106582 including 77 CRC patients was collected from Gene Expression Omnibus (GEO) database (https://www.ncbi.nlm.nih.gov/geo/) to identify DEmRNAs. In this study, the clinical information of 451 COAD patients was collected to further investigate the relationship between clinical characteristics and candidate genes based on TCGA, which is helpful to better understand the role of m6A modified circRNAs from the same host genes in the CRC progression. Clinical characteristics included pathological stage, survival status and overall survival time.

UALCAN (http://ualcan.path.uab.edu/index.html) is a comprehensive, user-friendly, and interactive web resource for analyzing cancer OMICS data (22). In this study, the protein expression of the potential prognostic genes were explored by the UALCAN tool based on CPTAC database. GEPIA2 (http://gepia2.cancer-pku.cn/#index) is a web-based tool to deliver fast and customizable functionalities based on TCGA and GTEx data (23). And multiple genes comparison analysis of the potential prognostic genes was performed by GEPIA. The cBioPortal for Cancer Genomics (http://cbioportal.org) provides a Web resource for exploring, visualizing, and analyzing multidimensional cancer genomics data (24). In our study, genetic alterations of the potential prognostic genes were analyzed by cBioPortal based on TCGA database. GeneMANIA (http://www.genemania.org) is a flexible, user-friendly web interface for generating hypotheses about gene function, analyzing gene lists and prioritizing genes for functional assays (25). And the co-expression information of the potential prognostic genes was analyzed by GeneMANIA. In the study, we paid more attention to TPM1 and analyzed the relationship between mRNA expression and the clinical factors of CRC (26), by adopting Wilcoxon test.

### Western Blotting

The total proteins of samples (including 4 CRC tissues and 4 paracancer tissues) were extracted, and the protein concentrations were determined by bicinchoninic colorimetric assay. Each tissue sample containing 30 ug of total proteins were separated through SDS-PAGE on a 10% gel. Subsequently, the separated proteins were transferred to the polyvinylidene difluoride (PVDF) membrane. The PVDF membrane was immunoblotted with TPM1 antibody (Abcam) at 4°C overnight, after blocking with 5% nonfat-dried milk. Then the PVDF membrane was incubated with corresponding secondary antibodies at room temperature for 1 hour. And TPM1 protein was visualized by enhanced chemiluminescence method. Finally, the PVDF membrane was immunoblotted with β-actin antibody (Beyotime) at 4°C overnight. And the PVDF membrane was incubated with corresponding secondary antibodies at room temperature for 1 hour. The enhanced chemiluminescence method was used to visualize the β-actin protein.

### Functional enrichment and protein-protein interaction analysis

In this study, 113 candidate genes whose circRNAs and mRNAs both were down-regulated in CRC, were performed functional enrichment analysis based on the Database for Annotation, Visualization and Integrated Discovery (DAVID, https://david.ncifcrf.gov/) (27). DAVID provides a comprehensive set of functional annotation tools for investigators to understand the biological meaning behind large lists of genes (28). In addition, Protein-Protein Interaction (PPI) analysis is valuable to investigate the molecular mechanisms of crucial cellular activities in tumorigenesis. And the STRING database (http://string-db.org/) was used to perform PPI analysis. The main parameters were set as follows, meaning of network edges (evidence), active interaction sources (Experiments), minimum required interaction score [low confidence (0.150)] and max number of interactors to show (no more than 50 interactors) (29).

## Results

### Screening of candidate genes

**Figure 1:**
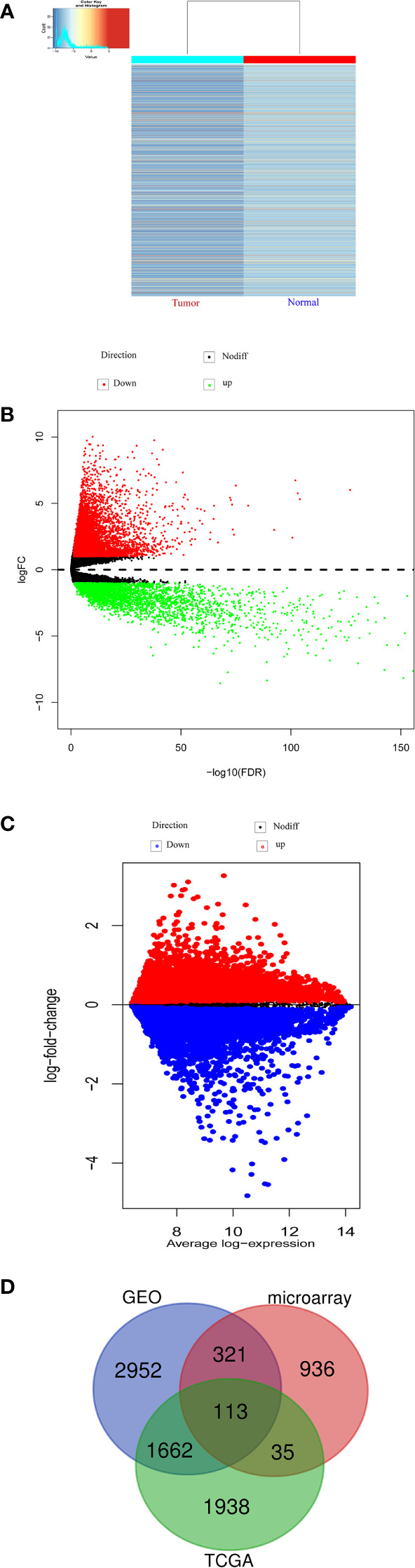
Differentially expressed m6A modified circRNAs and mRNA. **(A)** Hierarchical clustering heatmap of differentially m6A modified circRNAs. **(B)** Volcano plots of differentially mRNA based on TCGA-COAD. **(C)** Volcano plots of differentially mRNA based on GSE106582. **(D)** The intersected genes between microarray, COAD-TCGA and GSE106582.

With a threshold of |FC|>=2 and p-values<0.05, we found that 1405 significantly downregulated circRNAs in CRC by our microarray data. (**Table S1**). And cluster heatmaps indicates clustering of differentially m6A-methylated circRNAs between CRC and the control group (**Figure 1A**). In addition, a total of 506 COAD patients from TCGA database were explored DEmRNAs between CRC and the control group based on edgeR package. And we found that 12891 mRNAs were differentially expressed between CRC and the control group, including 3748 down-regulated mRNAs (**Figure 1B**). Similarly, we also found that 5048 mRNAs were down-regulated in the GSE106582 (**Figure 1C**). In this study, down-regulated mRNAs obtained from public database (**Table S2** and **Table S3**) were intersected with the gene symbol of down-regulated circRNAs from the same host genes to obtain the potential prognostic genes. And 113 candidate genes were obtained, as shown in **Figure 1D**.

### The prognostic analysis of CRC patients

**Figure 2:**
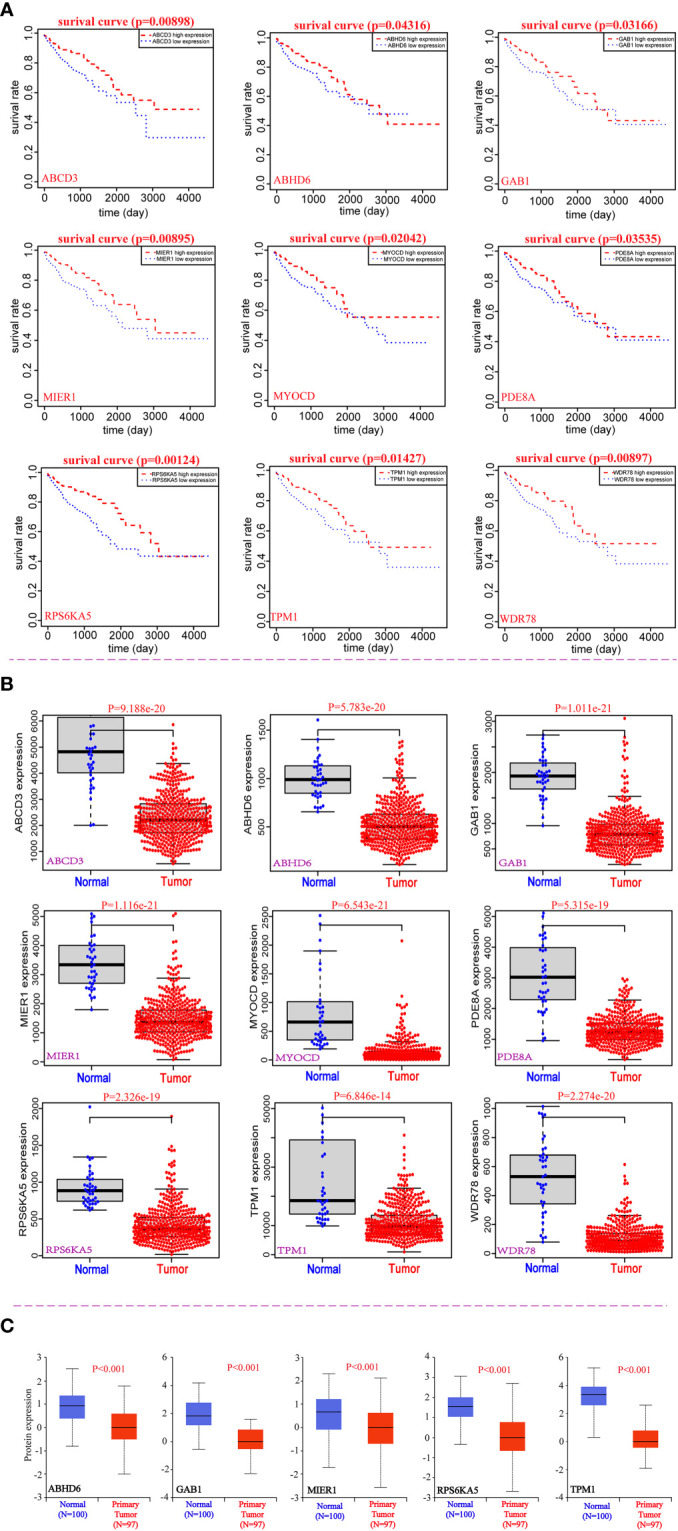
Correlation analysis between nine potential genes and the prognosis of CRC. **(A)** The prognostic value of nine potential genes in CRC. **(B)** The expression of nine potential genes at mRNA levels. **(C)** The expression of five potential genes at protein level.

In order to assess the clinical significance of the 113 candidate genes, the relationship between 113 DEmRNAs from the same host gens and the prognosis of CRC was explored by R packages based on TCGA database. We found that low expression of nine potential prognostic genes was significantly related to the poor prognosis of CRC patients (P<0.05), including *ABCD3*, *ABHD6*, *GAB1*, *MIER1*, *MYOCD*, *PDE8A*, *RPS6KA5*, *TPM1* and *WDR78* (**Table 1**). And the prognosis of overall survival of nine potential prognostic genes in CRC patients is presented in Kaplan-Meier curves (**Figure 2A**). In addition, we also found that the mRNA expression of nine potential prognostic genes were significantly lower in CRC patients than the control group (**Figure 2B**). Moreover, the protein expression of nine potential prognostic genes between CRC and the control group were further explored by the UALCAN tool based on CPTAC database. We observed that the protein expression of ABHD6, GAB1, MIER1, RPS6KA5 and TPM1 were significantly lower in CRC patients than the control group (**Figure 2C**).

**Table 1:** Information on circRNAs formed by nine parental genes.

circRNA_ID	Gene symbol	Regulation	Chromosome	circRNA_type
hsa_circRNA_100285	ABCD3	down	chr1	exonic
hsa_circRNA_103404	ABHD6	down	chr3	exonic
hsa_circRNA_406543	GAB1	down	chr4	exonic
hsa_circRNA_404525	MIER1	down	chr1	exonic
hsa_circRNA_042103	MYOCD	down	chr17	exonic
hsa_circRNA_036633	PDE8A	down	chr15	exonic
hsa_circRNA_405276	RPS6KA5	down	chr14	exonic
hsa_circRNA_035619	TPM1	down	chr15	exonic
hsa_circRNA_100259	WDR78	down	chr1	exonic

### Correlation between nine potential prognostic genes and pathological stages of CRC

**Figure 3:**
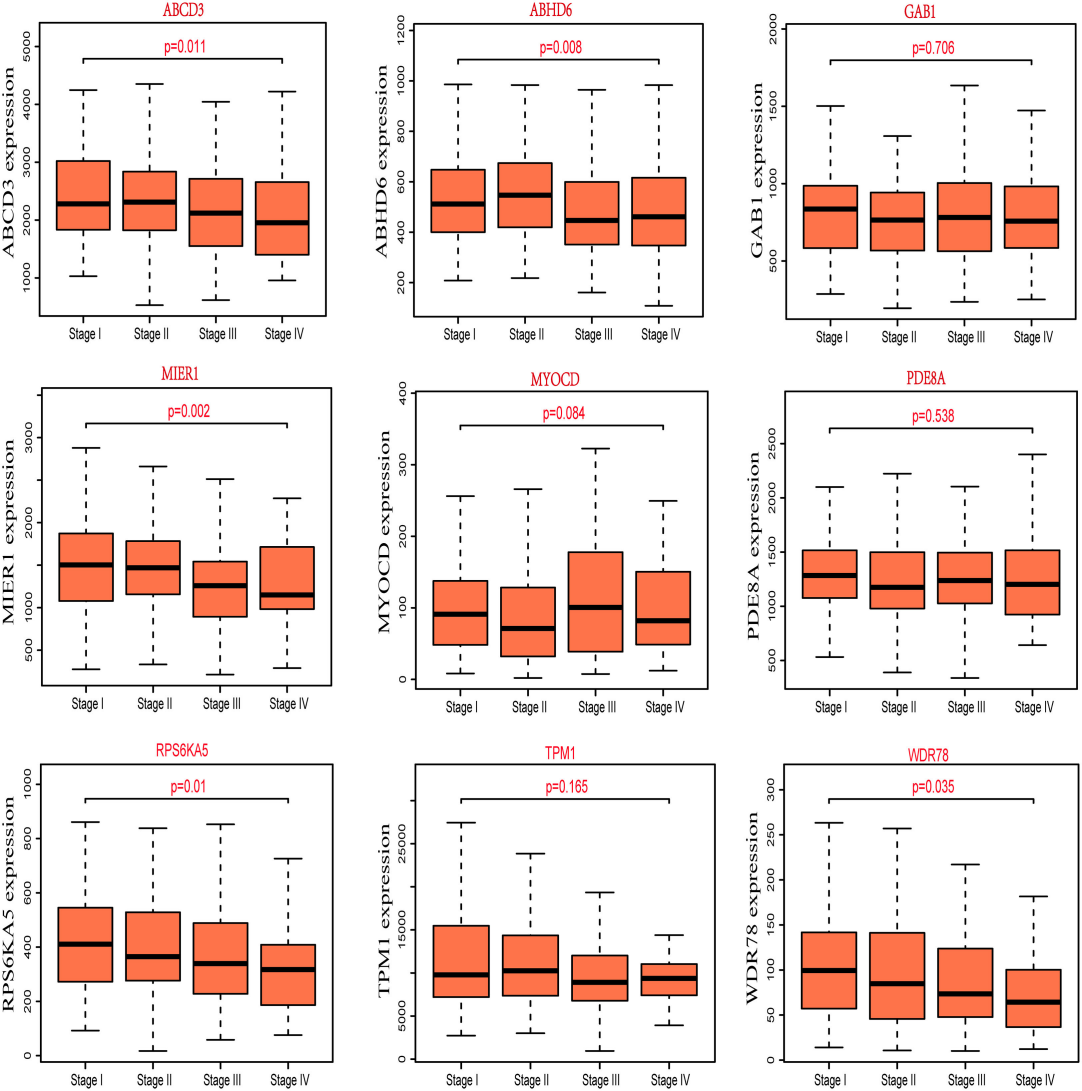
The expression of nine potential prognostic genes in different pathological stages of CRC.

For better to identify the progression of CRC patients, the expression of nine potential prognostic genes in different pathological stages was explored by R packages based on the clinical information of TCGA-COAD. We found that the correlation between expression of *ABCD3*, *ABHD6*, *MIER1*, *RPS6KA5* and *WDR78* and the different pathological stages of CRC was statistically significant (P<0.05), but not others (**Figure 3**). Additionally, we also observed that the expression of *MIER1*, *RPS6KA5* and *WDR78* decreases as the clinical stage of CRC patients. The result also suggested that these potential prognostic genes play a critical role in the progression of CRC.

### Relative expression, genetic alteration and co-expression of nine potential prognostic genes in CRC

**Figure 4:**
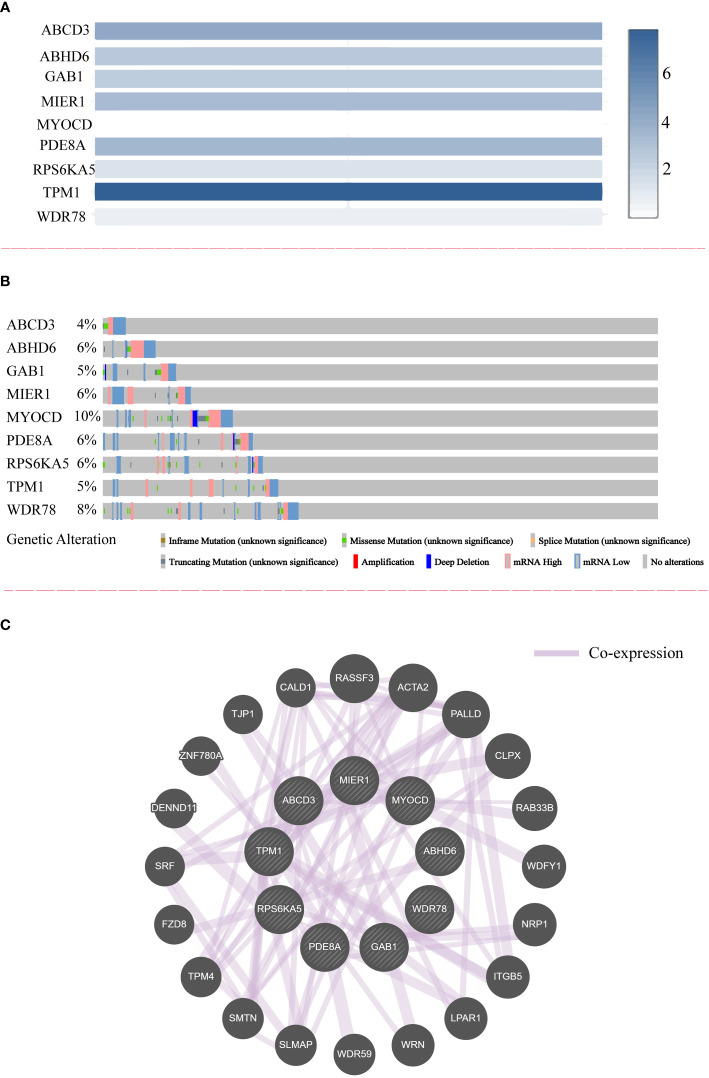
The relative expression, genetic alteration and co-expression of nine potential prognostic genes in CRC. **(A)** The relative expression of nine potential prognostic genes in CRC samples. **(B)** The genetic alteration of nine potential prognostic genes in CRC. **(C)** The co-expression network of nine potential prognostic genes.

In this study, the molecular characteristics of the potential prognostic genes were extensively analyzed. We compared the relative expression of nine prognostic genes in CRC and observed that the relative expression of TPM1 was the highest among all genes (**Figure 4A**). Additionally, the genetic alterations of nine potential prognostic genes in CRC were analyzed by the cBioPortal tool based on TCGA database. As shown in **Figure 4B**, *ABCD3*, *ABHD6*, *GAB1*, *MIER1*, *MYOCD*, *PDE8A*, *RPS6KA5*, *TPM1* and *WDR78* were altered in 4%, 6%, 5%, 6%, 10%, 6%, 6%, 5%, and 8% of the CRC samples respectively. At the same time, we found that the alteration frequency of *MYOCD* in CRC was highest among all potential prognostic genes (10% frequency). Finally, the co-expression network of nine potential prognostic genes was constructed by GeneMANIA (**Figure 4C**).

### Correlation analysis of TPM1 for clinical factors

**Figure 5:**
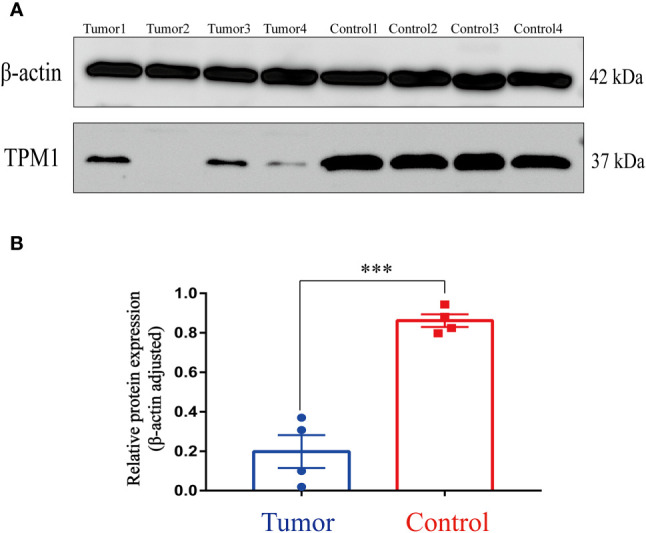
Western Blotting. **(A)** TPM1 protein expression in the tumor and control group. **(B)** ***P < 0.001.

In the study, we focused on the role of TPM1 in CRC, and it is more likely to be a reliable prognostic signature. We further analyzed the relationship between TPM1 mRNA expression and clinical factors of CRC by adopting Wilcoxon test, as shown in **Table 2**. We found that TPM1 mRNA expression was significantly related with age (p=0.045), gender (p=0.03), pathological stage (p=0.01) and lymph node metastasis (p=0.018), but not others. Additionally, we observed that the protein expressions of TPM1 in CRC were lower than the control group (**Figure 5A**). Subsequently, the blots were analyzed by Image J and GraphPad Prism. As shown in **Figure 5B**, we found that the protein expression difference between CRC and the control group was statistically significant (p=0.0003). Moreover, the uncropped strips are presented in **Figure S2**.

**Table 2:** Correlation analysis of TPM1 for clinical factors.

	*TPM1 expression*		Total	*p*-value
	High(n=191)	Low (n=191)		
Age				
<65 years	80(58.0%)	58(42.0%)	138	**0.045**
≥65 years	111(45.5%)	133(54.5)	244	
Gender				
Male	91(44.8%)	112(55.2%)	203	**0.03**
Female	100(55.9%)	79(44.1%)	179	
Pathological stage				
I-II	121(55%)	99(45%)	220	**0.01**
III-IV	70(43.2%)	92(56.8%)	162	
T classification
T1-T2	37(51.4%)	35(48.6%)	72	0.813
T3-T4	154(49.7%)	156(50.3%)	310	
Lymph node metastasis				
Negative	125(54.8%)	103(45.2%)	228	**0.018**
Positive	66(42.9%)	88(57.2%)	154	
Distant metastasis				
No	161(50.2%)	160(49.8%)	321	0.545
Yes	30(49.2%)	31(50.8%)	61	

Bold values indicate statistically significant clinical factors that are associated with the expression of TPM1 in colorectal cancer.

### Functional enrichment and protein-protein interaction analysis

**Figure 6:**
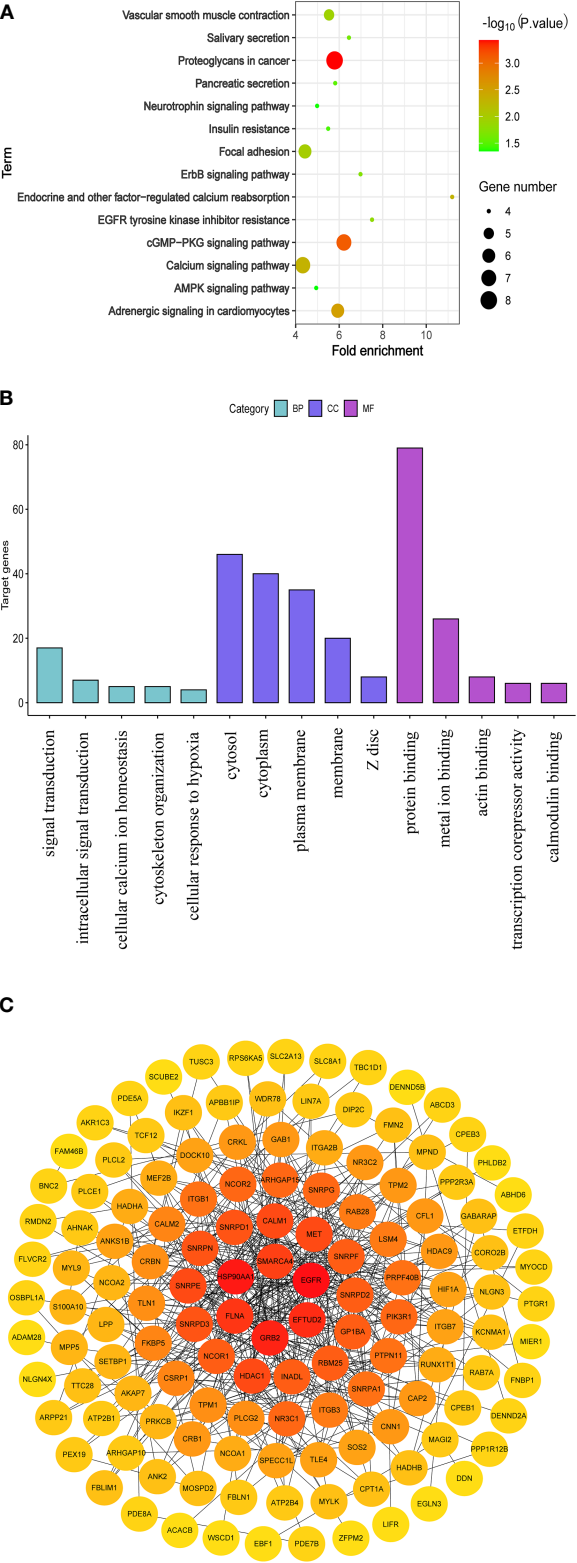
Functional enrichment and protein-protein interaction analysis of 113 parental genes for which both circRNAs and mRNAs were down-regulated. **(A)** KEGG pathway analysis. **(B)** GO analysis **(C)** The network of PPI.

Using the DAVID database, 113 candidate genes whose circRNAs and mRNAs both were down-regulated in CRC, were analyzed to explore the potential pathways and functions involved in the formation and progression of the CRC patients. And data visualization for enrichment analysis was performed by R packages. KEGG analysis suggested that 113 candidate genes were mainly enriched in proteoglycans in cancer, calcium signaling pathway, cGMP-PKG signaling pathway, adrenergic signaling in cardiomyocytes and focal adhesion (**Figure 6A**). Similarly, GO enrichment analysis was performed on 113 candidate genes, the result presented in **Figure 6B**. GO analysis suggested that all candidate genes were mainly enriched in signal transduction (GO term: BP), cytosol (GO term: CC), and protein binding (GO term MF). Additionally, the PPI analysis of 113 candidate genes was performed based on STRING database and the visualization was conducted by Cytoscape 3.8.2. As a result, several nodes of 165 and several edges of 501 were obtained in the PPI analysis (**Figure 6C**).

## Discussion

As one of the most common RNA modifications, m6A has been reported to involve the regulation of biological functions related to cancer and is closely associated with the occurrence, development, progression and prognosis of the patients with cancer (5, 30). In addition, growing evidence reveals that circRNAs contribute to various physiological and pathological processes, including the initiation and progression of cancer (31). It is worth noting that m6A modification also exists in the circRNAs and greatly affects the biological functions of circRNAs (32). Interestingly, some circRNAs located in the nucleus can regulate the transcription of the parental gene by binding to RNA polymerase II (33). Therefore, in order to better study the role of circRNAs in the pathogenesis of CRC, we pay more attention to explore the relationship between the parental genes of m6A-modified circRNAs and the progression of the patients with CRC.

A study revealed that circITGA7 and its linear host gene *ITGA7* are both significantly downregulated in colorectal cancer (CRC) tissues and cell lines (34). Interestingly, they proved that circITGA7 promotes the transcription of the parental gene *ITGA7* by suppressing the Ras signaling pathway and the low expression of both circITGA7 and *ITGA7* was correlated with the progression of CRC (34). Thus, it is necessary to explore the relationship between circRNAs and their parental genes in CRC. In this study, differentially m6A modified circRNAs of CRC were obtained by our microarray data. And differentially expressed mRNAs were identified based on the TCGA-COAD and GSE106582. In total, we found that 113 circRNA-mRNA pairs which were consistently downregulated between CRC and the control group. Then we analyzed the relationship between 113 candidate genes and the prognosis of CRC, and obtained nine potential prognostic genes, including *ABCD3*, *ABHD6*, *GAB1*, *MIER1*, *MYOCD*, *PDE8A*, *RPS6KA5*, *TPM1* and *WDR78*. And we speculate that the down-regulated circRNAs of these nine potential prognostic genes cause the low expression of their parental genes, which promote the progression of the CRC patients.

In this study, we screened the parental genes for which both circRNAs and mRNAs were down-regulated for KEGG enrichment analysis. The result has shown that 113 candidate genes were mainly enriched in cancer-related pathway, especially calcium-signaling pathway. Accumulating evidences have demonstrated that intracellular Ca^2+^ homeostasis is altered in cancer cells and the alteration is involved in tumor initiation, angiogenesis, progression and metastasis (35). It is noteworthy that a study has found that the aberrant expression of genes in calcium-signaling pathway can promote cancer cell proliferation, migration, and tumor metastasis (36). And we think that the calcium-signaling pathway may involve the process of the CRC progression.

Copy number variation is widespread in the human genome, including deletions, insertions, duplications, and complex multi-site variation ranging from 1000 bp to millions of bp (37). A variety of genetic alterations occur within the cancer genome during the tumorigenesis and progression of cancer (38). In this study, the characteristics of genetic alteration for nine potential prognostic genes are presented in **Figure 4B**. We observed high frequency of genetic alteration in nine potential prognostic genes, especially *MYOCD*, which are potential therapeutic targets of CRC.

## Conclusions

In a word, we identified nine potential prognostic genes, after analyzed the relationship between the parental genes of m6A modified circRNAs and the progression of CRC. Above all, our study further validated TPM1 can serve as a potential signature for CRC patients.

## Data availability statement

The original contributions presented in the study are included in the article/**Supplementary material**. Further inquiries can be directed to the corresponding authors.

## Ethics statement

The studies involving human participants were reviewed and approved by The Ethics Committee of the Second Affiliated Hospital of Guilin Medical College. The patients/participants provided their written informed consent to participate in this study.

## Author contributions

Conception and design: MO and MY; Collection and assembly of data: LD, WL, and XH; Development of methodology: CM, WC and JS; Data analysis and interpretation: LD and WL; Manuscript drafting: WL; Manuscript revision: MO, LD and WL. All authors read and approved the final manuscript.

## Funding

This research was funded by Guangxi Health Commission Key Laboratory of Glucose and Lipid Metabolism Disorders (grant no. 19-xkjs-05).

## Conflict of interest

The authors declare that the research was conducted in the absence of any commercial or financial relationships that could be construed as a potential conflict of interest.

## Publisher’s note

All claims expressed in this article are solely those of the authors and do not necessarily represent those of their affiliated organizations, or those of the publisher, the editors and the reviewers. Any product that may be evaluated in this article, or claim that may be made by its manufacturer, is not guaranteed or endorsed by the publisher.
