# Supplementary figures and images for: Microarray and bioinformatic analysis reveal the parental genes of m6A modified circRNAs as novel prognostic signatures in colorectal cancer
Source: Front Oncol. 2022 Jul 29;12:939790. doi: 10.3389/fonc.2022.939790 (PMC9373052; doi:10.3389/fonc.2022.939790)

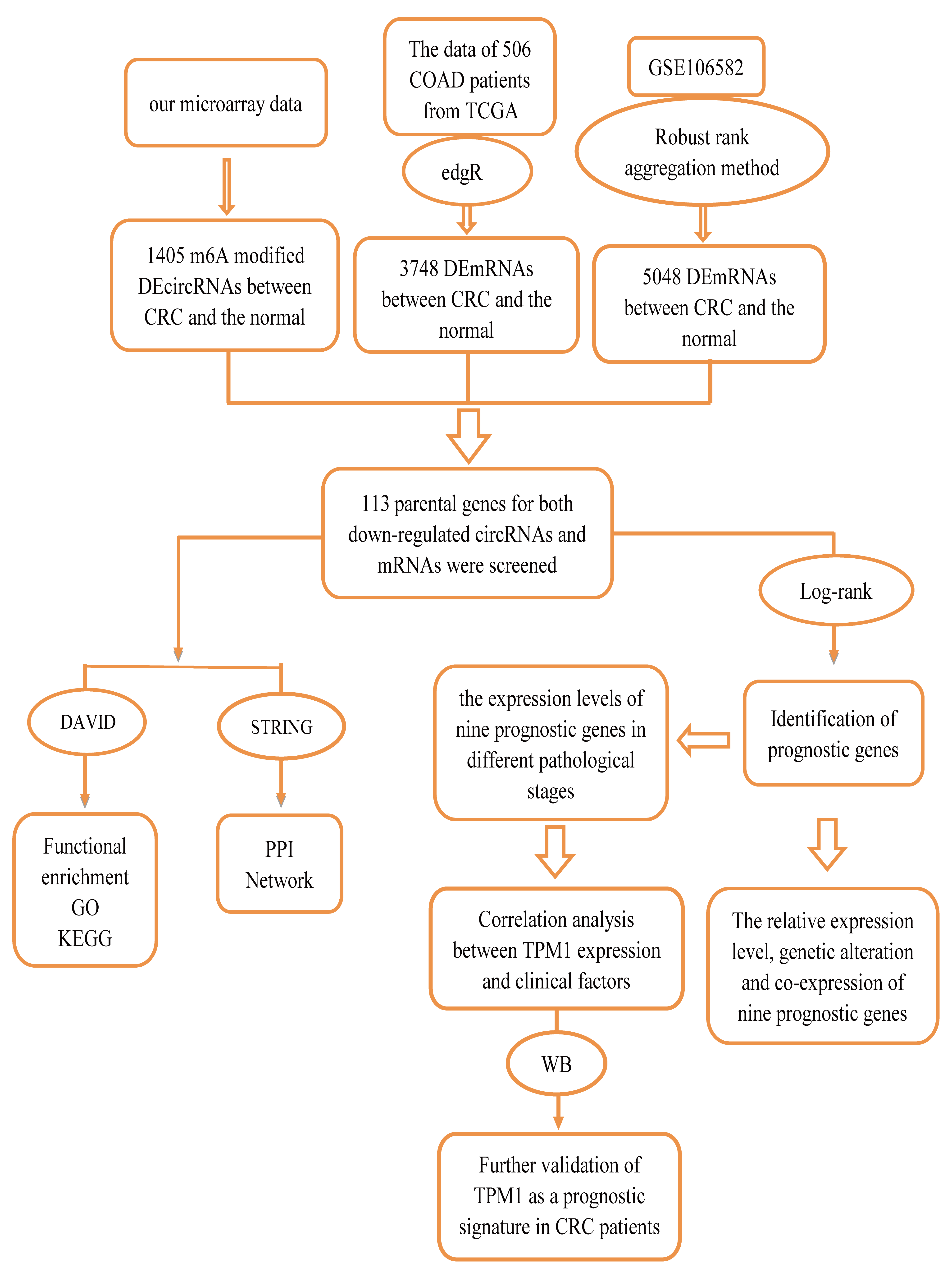

Supplement: Supplementary file 1 [file Image_1.tif]

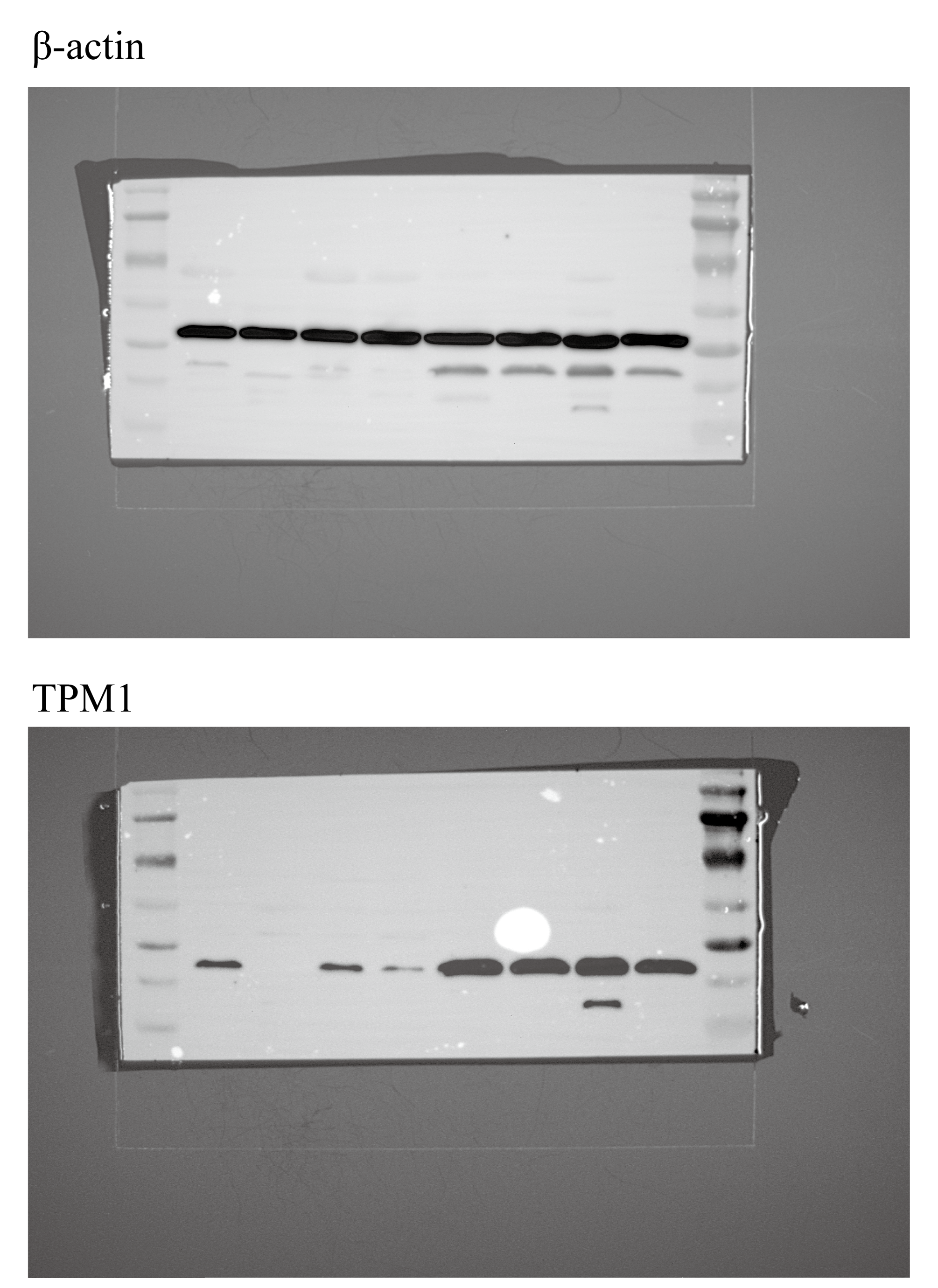

Supplement: Supplementary file 2 [file Image_2.tif]
